# Unlocking Syngas Synthesis from the Catalytic Gasification of Lignocellulose Pinewood: Catalytic and Pressure Insights

**DOI:** 10.1021/acssuschemeng.4c00320

**Published:** 2024-03-07

**Authors:** Kshitij Tewari, Sonit Balyan, Changle Jiang, Brandon Robinson, Debangsu Bhattacharyya, Jianli Hu

**Affiliations:** Department of Chemical and Biomedical Engineering, West Virginia University, Morgantown, West Virginia 26506, United States

**Keywords:** lignocellulose pinewood, catalytic gasification, high pressure, iron, nickel, syngas

## Abstract

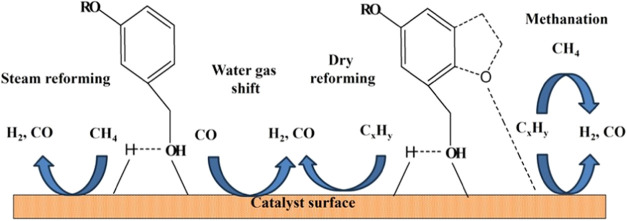

Modern technologies transform biomass into commodity chemicals, biofuels, and solid charcoal, making it appear as a renewable resource rather than organic waste. The effectiveness of Mo, Fe, Co, and Ni metal catalysts was investigated during the gasification of lignocellulosic pinewood. The primary goal was to compare the performance of iron and nickel catalysts in the low- and high-pressure production of syngas from pinewood. This is the first study that has reported high-pressure gasification of pinewood without the use of an external gasifying agent, producing syngas containing hydrogen, carbon monoxide, and carbon dioxide along with considerable amounts of methane with or without a catalyst. Also, the same gasification at low pressures was compared. In this study, the iron catalyst produces syngas more efficiently at higher pressure and 800 °C, and contains 43 mol % H_2_, 22 mol % CO_2_, 26 mol % CH_4_, and 8 mol % CO in comparison to the nickel catalyst. High pressure produces a large amount of methane too. The nickel catalyst produces higher syngas at low pressure and 850 °C, and contains 55 mol % H_2_, 9 mol % CO_2_, 5 mol % CH_4_, and 30 mol % CO. Low-pressure gasification produces less amounts of CH_4_ and CO_2_. Also, the H_2_/CO ratio is ∼1.81 using the nickel catalyst at low pressures, which is good for utilizing syngas as a feedstock. These results highlight the importance of catalyst selection, reactor configuration, and operating circumstances in adjusting gasification product composition. The study’s findings provide information about optimizing syngas production from pinewood, which is critical for the development of sustainable and efficient energy conversion technologies.

## Abstract 2

High-pressure gasification of pinewood combined with atmospheric pressure, without the use of an external gasifying agent, produced syngas.

## 1 Introduction

Biomass is a renewable energy source since it can be produced sustainably through methods such as reforestation, crop rotation, and waste-to-energy systems. Biomass gasification plays a crucial role in the development of renewable energy.^1^ Biomass gasification offers a means of mitigating greenhouse gas emissions in comparison with the utilization of fossil fuels. Although carbon dioxide (CO_2_) is indeed emitted during biomass combustion, it is essential to note that the carbon contained within biomass is an integral component of the natural carbon cycle. Consequently, the overall emissions resulting from biomass utilization are relatively lower, because the biomass feedstock actively absorbs CO_2_ during its growth phase. This action aids in the reduction of the impact of climate change. The utilization of trash: Biomass gasification can transform agricultural wastes, forestry waste, and various other organic materials into valuable energy resources. This process aids in mitigating the environmental consequences associated with waste disposal and the use of landfills.^2,3^ The utilization of syngas derived from biomass gasification offers a wide range of applications, encompassing power generation and heat production and serving as a feedstock for the manufacture of biofuels and chemicals. The inherent adaptability of this technology offers a range of possibilities for addressing energy requirements and presents itself as a viable option for energy storage.^3,4^ Biomass gasification is the conversion of biomass materials such as wood, crop leftovers, and other organic components into a gaseous fuel known as “syngas”. Syngas is predominantly composed of carbon monoxide (CO), hydrogen (H_2_), and minor quantities of methane (CH_4_) and carbon dioxide (CO_2_), accompanied by contaminants such as tars and particulate debris. This gaseous substance possesses versatile applications, encompassing the generation of heat and power, as well as the production of biofuels and chemicals.^5,6^

Diverse catalysts can elicit distinct impacts on the formation of syngas through the process of biomass gasification. The selection of catalyst is of utmost importance in influencing the composition, quality, and efficiency of the syngas generated. In previous studies, various catalysts have been used for biomass pyrolysis or gasification, in which the popular ones are nickel,^5,7−9^ iron,^10−12^ cobalt,^13^ molybdenum,^14,15^ cerium oxide,^16,17^ and various types of supports^18,19^ with these catalysts.

Catalytic biomass gasification is a complex procedure that involves multiple chemical processes, including pyrolysis, gasification, and the water–gas shift reaction. The primary area of interest for researchers lies in the development of catalysts with regard to the design of gasifiers and the specific characteristics of biomass feedstocks. In the present context, a catalyst that demonstrates exceptional performance in the removal of tar is seen to be very effective, as it ensures a gasification process that is both efficient and free from impurities. Additionally, the production of a syngas that possesses a composition specifically customized to accommodate the intended use is important. In addition, it is imperative for the catalyst to exhibit resistance against deactivation resulting from carbon fouling, thereby guaranteeing sustained operational efficacy over an extended period of time. The main features include cost effectiveness, strength, ease of regeneration, and compatibility with the reforming of syngas. Catalysts are of utmost importance in augmenting the efficacy and sustainability of biomass gasification, a crucial technique aimed at generating renewable energy and mitigating environmental repercussions.^20,21^

The utilization of syngas as a raw material for the synthesis of biofuels (such as bioethanol and biodiesel) and chemicals can be achieved by subsequent processing methods, including the Fischer–Tropsch synthesis.^22^ The production of hydrogen involves the generation of syngas that is rich in hydrogen content. This hydrogen-rich syngas can serve as a sustainable fuel source or be used as a raw material for various hydrogen-dependent industrial processes. The creation of syngas through the process of biomass gasification presents the benefit of transforming sustainable biomass resources into flexible energy carriers and chemical precursors. Nevertheless, achieving high efficiency and meeting specified quality standards for the syngas necessitate meticulous control of gasifier settings, effective impurity management, and process optimization. The efficiency and environmental performance of biomass gasification to syngas generation processes are being enhanced by ongoing research and development efforts in this field.^23−25^ Syngas is a widely used raw material in the chemical and industrial sectors, providing a versatile raw material for a variety of essential industrial processes and playing an increasingly important role in meeting energy demand and sustainability goals. Currently, around 50% of the global production of syngas is used in the production of ammonia, while the remaining 25% is used to produce hydrogen, and the rest of the syngas can also be transformed into a variety of other products, such as methanol and Fischer–Tropsch (FT). On an annual basis, syngas production amounts to approximately 6 Exajoules (EJ), which accounts for approximately 2% of the global energy consumption.^26,27^

Previous studies reported that LD Slag (LD slag is “steel conversion slag”) as an oxygen carrier in a 1.5 kWth unit analyzes catalytic gasification in the context of biomass chemical looping for syngas synthesis. They explored the function of LD Slag as a catalyst and looked at how it affects the chemical looping gasification reactions. Their study intended to improve the sustainability and efficiency of syngas generation from biomass by using LD Slag as an oxygen carrier, producing 25 vol % H_2_, 29.2 vol % CO, 33.4 vol % CO_2_, and 10.8 vol % CH_4_ as shown in Table 3.^28^ Another study focused on the use of a continuous unit and a synthetic Fe_2_O_3_/Al_2_O_3_ oxygen carrier in biomass chemical loop gasification, specifically in the context of catalytic gasification for syngas production. Their study advanced our knowledge of and ability to use catalytic gasification technologies, particularly as it relates to the efficient and sustainable synthesis of syngas from biomass using synthetic oxygen carriers. They reported syngas conversion (32 vol % H_2_, 37 vol % CO, 25 vol % CO_2_, and 9.5 vol % CH_4_) using 20 wt % Fe_2_O_3_/Al_2_O_3_ as a catalyst at 940 °C as reported in Table 3.^29^ Gasification is a potential technique for producing biopower and biofuels from biomass waste; this study uses a bench-scale fluidized bed reactor to gasify switchgrass and pine wastes using both air and air steam. Under constant operating conditions, the study examines how bed materials (sand, CaO + sand, Al_2_O_3_, and CaO + Al_2_O_3_) and biomass type affect the gasification products. A thorough analysis shows that the mix of biomass has a major impact on the output of CO (20.2 vol %) and H_2_ (21.1 vol %). CaO increases the H_2_ concentration by promoting CO_2_ carbonation and water–gas shift processes. Increased H_2_ concentration from adding more steam along with CaO results in high-quality syngas with less tar and contaminating gases.^30^

In the present study, we tested several catalysts with lignocellulose pinewood, including Mo, Fe, Co, and Ni. We focused on the reaction mechanism with respect to the pressure effects of the syngas compositions. We investigated iron and nickel catalysts for synthetic gas generation from pinewood based on comparison. The gasification data analysis was performed at various temperatures and low and high pressures without the use of an external gasifying agent. Pinewood contains 7.47 wt % moisture and has 46.66 wt % O content. Here, oxygen and steam used as gasifying agents act as limiting reactants. We produced syngas using iron at high pressures, resulting in a significantly high amount of methane due to the pressurized effect, which would be further converted into syngas by steam methane reforming. We obtained hydrogen-rich syngas at low pressures by utilizing a nickel catalyst with low methane and carbon dioxide contents. The key factors in this work are as follows: (1) no external gasifying agent was used (effect: oxygen used as a limiting reactant); (2) the pressurized effect produces a high concentration of methane in syngas; (3) low pressure produces a rich syngas concentration with low amounts of CH_4_ and CO_2_.

## 2 Experimental Section

### 2.1 Materials

In this experimental study, all chemicals and reagents used were of analytical grade. All of them are of high-purity grade. Pinewood (Lowe’s), nickel(II) nitrate hexahydrate (99%) (Acros Organics), ferric nitrate nonahydrate (98.6%) (Fisher Scientific), cobalt(II, III) oxide (99.7%) (Alfa Aesar), and molybdenum powder (99.9%) (Alfa Aesar) were purchased. After all of the reagents were received, no further purification was required. Tedler Gas bags (Thermo Scientific) (1 L) were used for sample injection for the Micro Gas Chromatograph (Inficon Inc.). Deionized water was used for the preparation of all aqueous solutions.

### 2.2 Catalyst Preparation

Nickel(II) nitrate hexahydrate and ferric nitrate nonahydrate were dissolved in deionized water in a 100 mL beaker at 350 rpm and room temperature. The samples were dried at 120 °C for 4 h, followed by calcination in air for 400 °C for 2 h to convert metal nitrates to oxides. Thereafter, they were dried at 120 °C for 4 h in an oven. After that, both iron and nickel dried samples were subjected to calcination and reduction processes and their characterization is shown in Figures S3 and S4. For calcination, both samples were filled one by one in a 0.5 in. quartz tube, packed with glass wool, heated at a ramp rate of 10 °C/min until reaching up to 400 °C in the presence of N_2_ gas at 50 sccm/min, and held for 2 h. In the calcination process, ferric nitrate and nickel nitrate were converted into iron oxide and nickel oxide, respectively. Thereafter, the temperature was increased up to 650 from 400 °C at 10 °C/min in the presence of H_2_ gas at 50 sccm/min and held for 2 h for converting iron oxide into iron. Similarly, the temperature was increased up to 450 from 400 °C at 10 °C/min in the presence of H_2_ gas at 50 sccm/min and held for 2 h for converting nickel oxide into nickel, and both were used for further reactions. Other catalysts such as molybdenum powder, and cobalt oxide were used directly for the reaction.

### 2.3 Reactor Design

**Figure 1:**
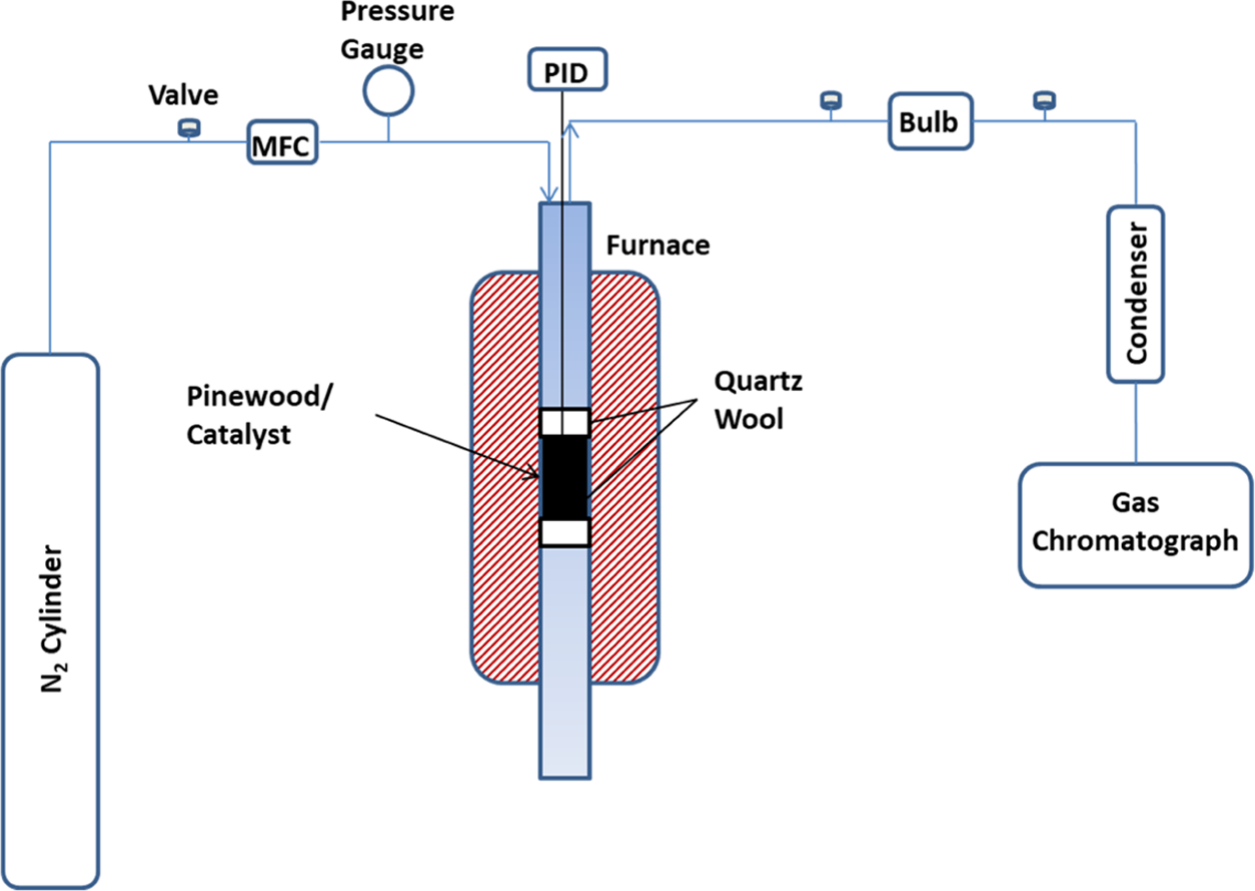
Schematic of Pinewood Gasification.

Lignocellulose pinewood gasification was performed in a fixed-bed reactor (10.5 mm internal diameter, 12.7 outer diameter, and 381 mm long cylindrical tube) made of stainless steel 316 SS tube (Charlestin Valve and Fitting) shown in Figure 1. For all experimental runs, we optimized the amount of catalyst and pinewood for this reactor tube. Typically, 1.0 g of lignocellulose pinewood and 0.15 g of the catalyst were used for all low-pressure and high-pressure reactions. All low-pressure reactions were run at 1.01 bar or atmospheric pressure and high-pressure reactions were run at 29 to 38 bar.

In the present experimental configuration, the biomass underwent meticulous processing using grinding and screening procedures to get a mean particle diameter of 200 μm (∼150–250 μm). The selection of this particle size was made in order to ensure uniformity in the particles employed in the experimental investigation of a fixed-bed reactor at a smaller scale. We used a mean particle diameter of 200 μm to build a bubbling fluidized bed for an upcoming project. This means that the particle diameter is good for fluidization. This system is presently being subjected to testing under different parameter configurations. The factors encompassed in this study comprise temperature, pressure, no gasifying agent, and a feed/catalyst material ratio. The experimental procedure involved conducting high-temperature reactions in a Carbolite furnace, USA that was equipped with a programmable controller. In order to measure the temperature of the reactor bed, a K-type thermocouple (OmegaTM) was embedded in the reactor with PID as shown in Figure 1.

### 2.4 Elemental Analysis of Feedstock

Ultimate and proximate analyses of the lignocellulose pinewood are shown in Table 1. In Table 1, the utilization of proximate analysis is crucial in evaluating the composition of biomass, which involves the determination of parameters such as moisture content, volatile matter, fixed carbon, and ash. The moisture content plays a crucial role in the management and processing of biomass, impacting the efficiency of transportation and the total conversion procedures. The presence of volatile matter has a substantial influence on the process of thermal conversion as it leads to the production of a greater quantity of combustible gases. Nonetheless, the inclusion of ash, an inorganic constituent included in biomass, can result in adverse effects such as fouling, agglomeration, and slagging in reactors, furnaces, and associated apparatus. Conversely, the ultimate analysis investigates the elemental constitution of biomass, with particular emphasis on fundamental components such as carbon (C), hydrogen (H), nitrogen (N), and sulfur (S). The determination of oxygen (O) content was conducted by an indirect approach, whereby it was derived by subtracting the sum of the percentages of other elements from 100%. This detailed research facilitates comprehension of the fundamental attributes of the biomass, hence providing guidance for its efficient consumption and processing in diverse energy conversion systems.

**Table 1:** Pinewood Biomass Elemental Composition (Obtained from the National Research Center for Coal and Energy Certified for Standard Testing, WVU)

ultimate analysis					
element	C	H	N	S	O
wt %	48.478	5.546	0.312	0	45.664

proximate analysis				
parameter	moisture	volatile	fixed carbon	ash
wt %	7.47	74.4	18.075	0.055

### 2.5 Gas Product Analysis

First, prior to starting the reaction in the reactor, the catalyst was combined with the biomass and introduced into the fixed-bed reactor in a weight proportion of 0.15:1. Thereafter, we passed the nitrogen gas consisting of 200 standard cubic centimeters per minute (sccm) for 30 min to create an oxygen-free environment. The reactor was initially supplied with a nitrogen gas consisting of 100 sccm of nitrogen for 6 min, resulting in an initial pressure of 1.5–2 bar in a pressure gauge. The target reaction temperature was attained by gradually raising the temperature of the bed at the rate of 10 °C per minute, followed by maintaining it for a duration of half an hour. Thereafter, for pressurized reactions, before increasing the temperature of the reactor, the inlet (of nitrogen gas) and outlet valves of the reactor were closed as shown in Figure 1. The reaction conditions for high pressure have been exhibited at three different temperatures, 750, 800, and 850 °C, and the pressure of the reactor reached up to 38 bar. The reactor pressure exhibited variations depending on the temperature of the reaction, with values of 750–900 °C with corresponding pressures in-between 28–38 bar. After completing the reaction, the gas sample was collected in a SKC Tedlar 1L sample bag and thereafter analyzed by micro GC. But for low-pressure or atmospheric pressure reactions, the outlet valve of the reactor was opened after creating an oxygen-free environment inside the reactor. Here, the gas samples were collected and analyzed continuously from the starting of the reaction at 3 min intervals.

Gas samples were procured for the purpose of product analysis at the specific reaction temperature by employing SKC Tedlar 1 L sample bags. The samples were later subjected to analysis using a gas chromatograph, namely the Inficon Fusion model and PerkinElmer Clarus 690. The diagram depicting the configuration of the fixed-bed gasifier reactor setup is presented in Figure 1. Gas chromatography equipment was utilized to analyze the product gases obtained from lignocellulose pinewood gasification studies conducted in a fixed-bed reactor at different temperatures and pressures. The Inficon Fusion micro GC employed a configuration consisting of four columns. The four columns were equipped with distinct characteristics: a molecular sieve column including a 3-m-long PLOT U precolumn, an 8-m-long RT-PLOT U column accompanied by a 1-m-long PLOT Q precolumn, an 8-m-long aluminum column, and a 20-m-long RTX-1 column. This configuration facilitated precise detection at the parts per billion (ppb) concentration of diverse gases, including H_2_, CH_4_, CO, CO_2_ (enabling a comprehensive syngas analysis), C2–C4 hydrocarbons like ethylene, ethane, acetylene, as well as water and nitrogen (N_2_). In order to examine the constituents of pinewood gasification, such as char and other substances, the PerkinElmer Clausius 690-GC instrument was employed for the purpose of detecting tar contamination or liquid products. The gases employed for calibration in this study were sourced exclusively from ultrahigh purity grade, hence guaranteeing the analytical data accuracy and precision.

## 3 Results and Discussion

### 3.1 Biomass Gasification and Its Chemical Reactions in a Fixed-Bed Reactor

Gasification turns solid biomass into a gaseous mixture containing carbon monoxide (CO), hydrogen (H_2_), carbon dioxide (CO_2_), methane (CH_4_), organic vapors, tars such as benzene, and other aromatic hydrocarbons, some of which are also vaporized, water vapor, residual solids, and trace elements. The basic engineering and reactions during biomass gasification are explained below. During the biomass gasification process, lignocellulose biomass is converted into volatile gases, char, and tar, as shown in the equation gasification reaction (G1).
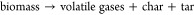
G1

The composition of this gaseous mixture varies based on several parameters, including the conditions of gasification and the prevailing ambient conditions, in the presence of inert. Furthermore, gasification has the ability to convert the inorganic materials present in biomass into ash, including silicon (Si), aluminum (Al), titanium (Ti), iron (Fe), calcium (Ca), magnesium (Mg), sodium (Na), potassium (K), phosphorus (P), sulfur (S), and chlorine (Cl). This extensive change emphasizes the diverse character of gasification, as well as its potential importance in sustainable energy production and waste management. Understanding the complex interplay of these elements is critical for improving the gasification process and realizing its potential in a variety of industrial and environmental applications.

Biomass gasification comprises a series of discrete thermal processes. When heated, biomass passes through a drying phase that lasts until the temperature reaches around 120 °C. As the temperature climbs to roughly 350 °C, in this temperature range, oxidation G2 and partial oxidation reaction G3 take place, which are highly exothermic; from this temperature range, the volatile components of the biomass are released via a devolatilization process. Beyond this point, the residual solid material, known as char, is gasified. The process of bond dissociation in lignin molecule and how it converts into syngas is described in Supporting Information (SI). As a result, the gasification process is typically separated into four steps: drying, devolatilization, gasification, and tar conversion.
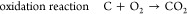
G2
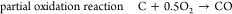
G3

Gasification is a combination of pyrolysis and oxidation processes. The chemical components in biomass are subjected to temperatures ranging from 500 to 900 °C without the presence of a gasifying agent. In this temperature range, mainly three gasifying reactions G4–G6 happen along with homogeneous volatile reactions G7–G11 and tar conversion reactions G12–G16.
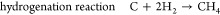
G4
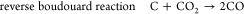
G5
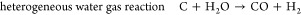
G6

The heat required to power these reactions can be either from outside sources or created within the gasifier unit itself, most commonly by exothermic combustion of biomass. This complex gasification process is essential to the use of biomass as a sustainable energy source and has promise for a variety of industrial uses.
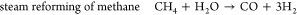
G7
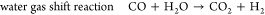
G8
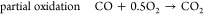
G9
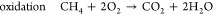
G10
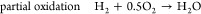
G11

Evans and colleagues reported that when the temperature of the gasification process rises, the composition of the tars changes. It proceeds in a precise order, beginning with mixed oxygenates and progressing through phenolic ethers, alkyl phenolics, heterocyclic ethers, polyaromatic hydrocarbons, and finally, bigger polyaromatic hydrocarbons. This series of modifications illustrates the changing nature of the chemical reactions that occur as the temperature rises. An analysis of high-temperature tars generated from cellulose reveals the presence of levoglucosan as a main component.^31^
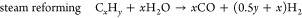
G12
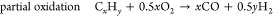
G13
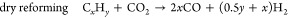
G14
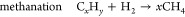
G15
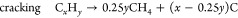
G16

Furthermore, the period of gasification is important in the tar conversion. Longer vapor residence periods at pyrolysis temperatures result in greater secondary vapor-phase cracking, leading to the production of extra gases, water, and low-molecular-weight compounds. One of the byproducts of biomass gasification, known as char, retains the structural properties of the original lignocellulosic material. The pyrolysis temperature influences the rate of char generation, with slower pyrolysis processes producing more char. However, as the temperature increases, the char yield reduces dramatically, especially until it reaches around 400 °C. The char undergoes a change as the temperature increases, becoming increasingly fragrant and carbon rich. The elimination of hydroxyl, aliphatic, carbonyl, and olefinic C=C groups is responsible for this alteration. Furthermore, at higher gasification temperatures, the release of volatile materials causes voids within the pore structure of the char. Additional temperature increases can cause char softening, melting, and fusing, with a notable contraction in the carbon structure occurring beyond 500 °C, corresponding to the aromatization process.^32,33^

### 3.2 Biomass Noncatalytic Gasification in a Fixed-Bed Reactor

**Figure 2:**
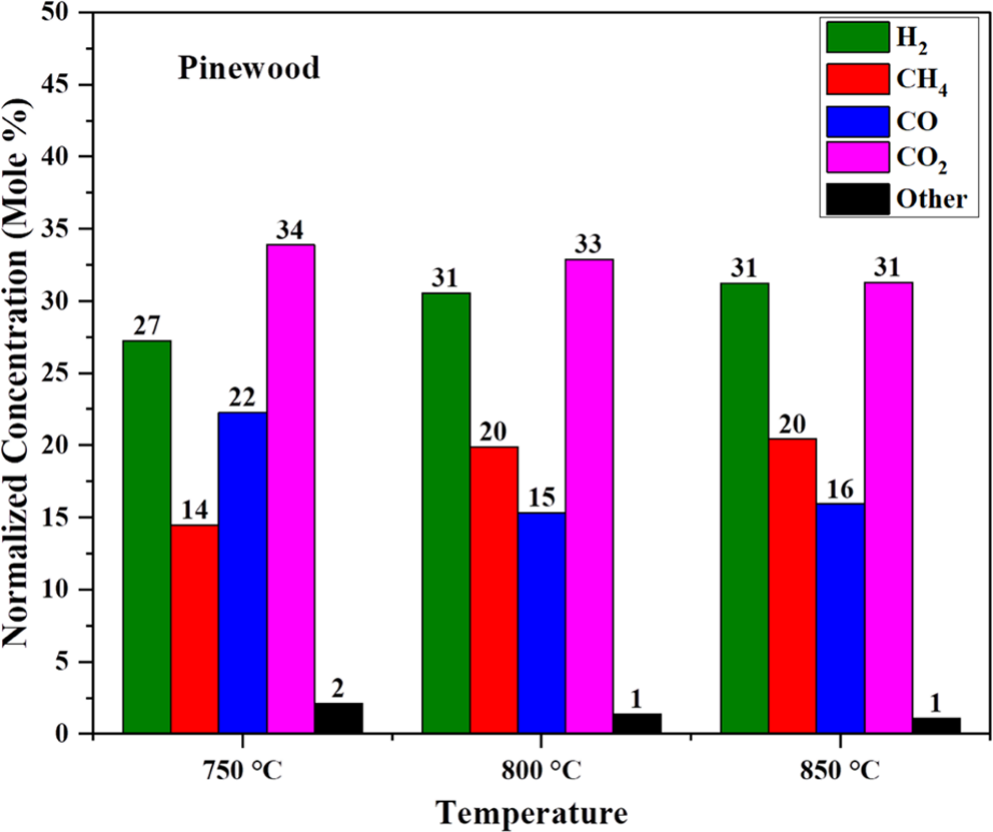
Effect of temperature on gasification of PW for syngas production (pressure: 28–38 bar).

Figure 2 shows the normalized concentration of syngas for noncatalytic lignocellulose pinewood gasification at different temperatures. The gas, char, and tar yield from the pinewood gasification are shown in Figure S1. The reaction conditions for this comparison are as follows. Three different temperatures were tested: 750, 800, and 850 °C, respectively, shown in Table 2. The ramp rate was 10 °C/min and reaction time was 30 min. The pressure was 29, 33, and 38 bar, respectively. It was observed that increase in reaction temperature increases hydrogen production as well as methane production due to the high-pressure effect. The syngas concentration at 750 °C was reported as 27.23 mol % H_2_, 14.47 mol % CH_4_, 22.27 mol % CO, 33.87 mol % CO_2_, and 2.13 mol % others and the syngas concentration at 800 °C was 30.55 mol % H_2_, 19.88 mol % CH_4_, 15.31 mol % CO, 32.87 mol % CO_2_, and 1.37 mol % others. The syngas concentration at 850 °C was reported as 31.24 mol % H_2_, 20.43 mol % CH_4_, 15.93 mol % CO, 31.28 mol % CO_2_, and 1.09 mol % others.

**Table 2:** Syngas Production from Lignocellulose Pinewood Data at Low and High Pressures

	pressure (bar)	temperature (°C)	H_2_ (mole %)	CH_4_ (mole %)	CO (mole %)	CO_2_ (mole %)	H_2_/CO
PW	28	750	27.23	14.47	22.27	33.87	1.22
	32	800	30.55	19.89	15.31	32.88	2.00
	38	850	31.24	20.44	15.93	31.29	1.96
PW/Fe	30	750	31.04	11.22	22.07	34.80	1.41
	34	800	43.07	26.13	7.67	21.54	5.61
	38	850	36.50	21.04	15.74	24.71	2.32
PW/Ni	30	750	20.57	33.25	14.58	30.74	1.41
	34	800	21.59	29.85	18.39	28.49	1.17
	38	850	28.43	14.40	25.83	29.66	1.10
PW	1.01	750	34.83	22.87	18.26	21.49	1.91
	1.01	800	36.23	12.49	24.81	24.51	1.46
	1.01	850	39.24	8.25	28.72	22.20	1.37
PW/Fe	1.01	750	39.67	12.05	24.98	21.67	1.59
	1.01	800	42.32	7.70	39.67	9.76	1.07
	1.01	850	47.19	7.32	34.21	10.28	1.38
PW/Ni	1.01	750	44.88	7.52	29.41	17.07	1.53
	1.01	800	48.10	8.49	29.10	13.28	1.65
	1.01	850	54.99	4.95	30.30	9.10	1.81

**Figure 3:**
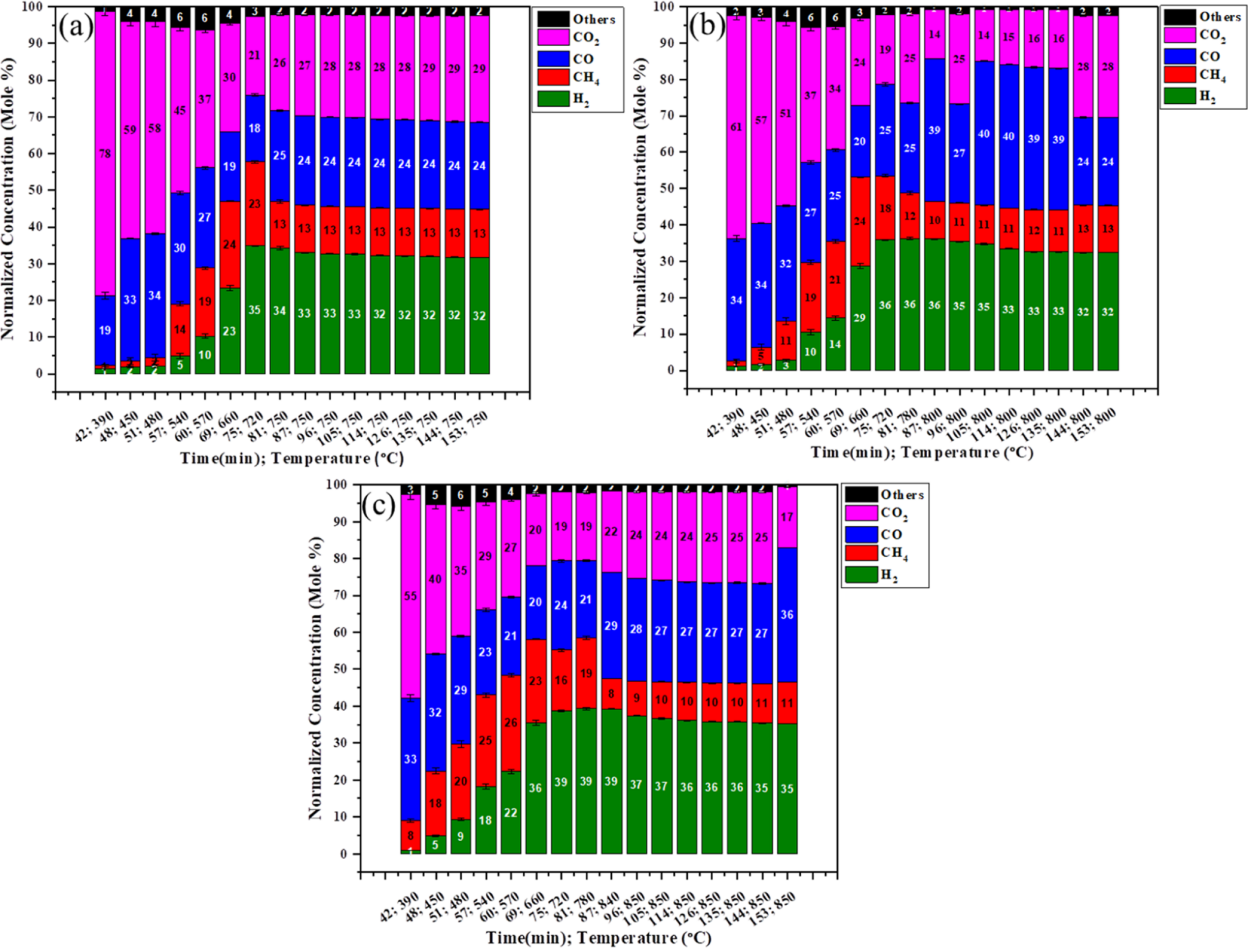
Effect of PW for synthetic gas production at low pressure and 750 °C (a), 800 °C (b), and 850 °C (c).

Figure 3a–c shows the lignocellulose biomass gasification at low pressure at different temperatures (750 °C, 800 °C, and 850 °C) with respect to time as shown in Table 2; the detailed gas, char, and tar yields from the pinewood gasification are shown in Figure S2. Initially, when the temperature is greater than 380 °C the combustion reactions G2 and G3 readily happen; until 380 °C, max concentrations of CO_2_ and CO are achieved. Above this temperature, the hydrogenation G4 and heterogeneous water gas reaction G6 occur with production of H_2_ and CH_4_. The absence of a gasifying agent mandates additional energy for the dissociation of C–C, C=C, and C–H bonds, resulting in higher temperatures being required. Without a catalyst gasifying agent, the water gas shift reaction will happen but to a very low extent, as it is not a rate-determining step. At 750 °C, with increase in temperature, the concentrations of CO_2_ and CO decrease as time increases and the concentrations of CH_4_ and H_2_ increase with time. Here, an increase in temperature causes the homogeneous volatile reactions to take place in which the system undergoes the steam methane reforming G7 and water gas shift reaction G8 to very little extent. Tar reactions G12–G16 happen at greater than 700 °C. The syngas concentration at this temperature comprises 34 mol % H_2_, 22.8 mol % CH_4_, 18.2 mol % CO, 21.5 mol % CO_2_, and 1.9 mol % others. At 800 °C, the only changes after increasing the temperature are a slight decrease in CO_2_ concentration and increase in CO concentration; also, partial dry reforming G14 happens to a limited extent and the syngas concentration comprises 36.2 mol % H_2_, 12.4 mol % CH_4_, 24.8 mol % CO, 24.5 mol % CO_2_, and 2.54 mol % others. At 850 °C, the only change found is that after 81 min, at 760 °C the methane conversion decreases with time due to the stream forming of methane G7 occurring. The syngas concentration comprises 39.2 mol % H_2_, 8.25 mol % CH_4_, 28.71 mol % CO, 21.66 mol % CO_2_, and 1.63 mol % others.

### 3.3 Comparison of Various Catalytic Biomass Gasification Processes in a Fixed-Bed Reactor

The selection of a catalyst is contingent on the particular objectives of the gasification procedure and the intended composition of the syngas. The selection of a suitable catalyst is influenced by various factors, including the kind of feedstock, the design of the gasifier, the operating circumstances, and the planned application of the syngas. Furthermore, the gradual loss of catalyst activity over time and the procedures employed to restore catalyst functionality are also crucial factors to be taken into account in the whole process. Researchers and engineers persistently engage in the development and enhancement of catalysts for diverse gasification applications with the aim of enhancing efficiency and mitigating environmental consequences.^34^

**Figure 4:**
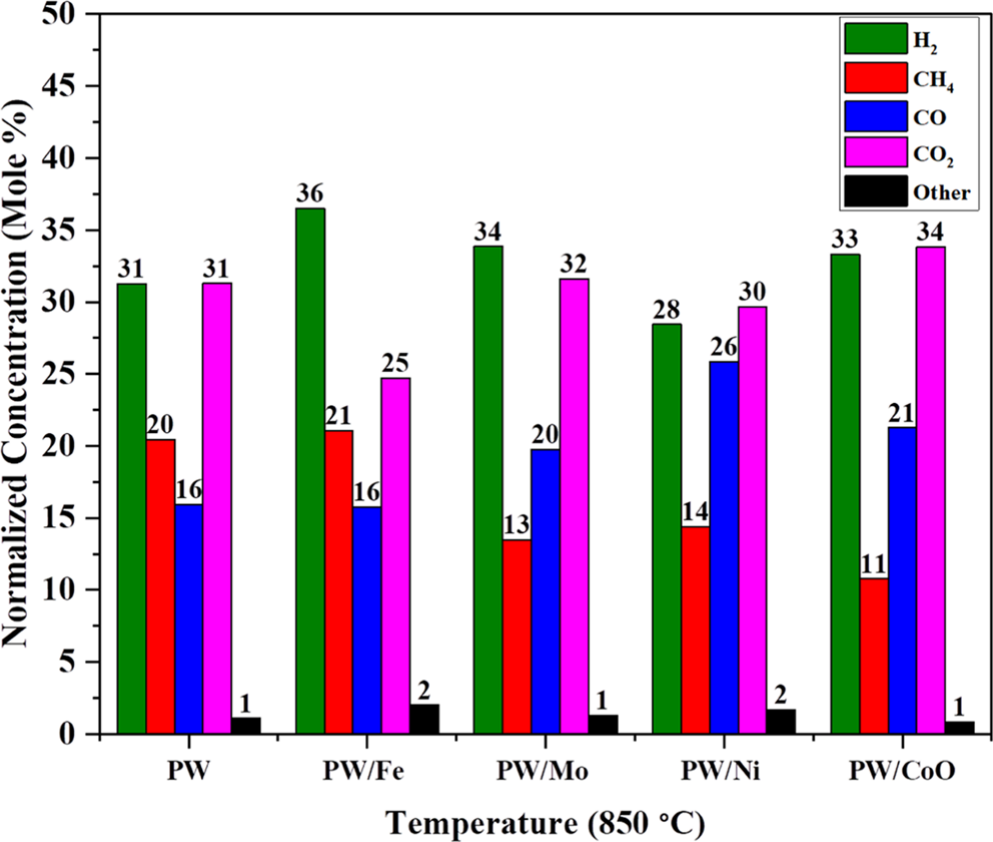
Synthetic gas production with or without catalyst at high pressure (process conditions: temperature: 850 °C, pressure: 35 bar).

Based on previous studies,^5,15,20^ the popular catalysts used for biomass pyrolysis or gasification are nickel, iron, cobalt, and molybdenum. The basic characteristics like surface area and average particle size of the catalyst are shown in Table S1. Figure 4 shows the normalized concentration of syngas for noncatalytic and catalytic lignocellulose pinewood gasification. The optimized reaction conditions for this comparison are as follows: reaction temperature 850 °C, ramp rate 10 °C/min, residence time 30 min, and pressure 30–38 bar. In this study, biomass catalytic gasification was performed between the four popular catalysts: iron, nickel, molybdenum, and cobalt oxides at high pressures. The syngas normalized concentrations (mole %) for H_2_, CH_4_, CO, CO_2_, and other products for lignocellulose pinewood are 31.24, 20.44, 15.93, 31.29, and 1.09%, respectively; for lignocellulose pinewood with iron catalyst, syngas normalized concentrations (mole %) for H_2_, CH_4_, CO, CO_2_, and other products are 36.49, 21.04, 15.74, 24.71, and 2.00%, respectively. For lignocellulose pinewood with molybdenum catalyst, the syngas normalized concentrations (mole %) for H_2_, CH_4_, CO, CO_2_, and other products are 33.85, 13.48, 19.73, 31.62, and 1.30%, respectively; for lignocellulose pinewood with nickel catalyst, the syngas normalized concentrations (mole %) for H_2_, CH_4_, CO, CO_2_, and other products are 28.42, 14.39, 25.83, 29.65, and 1.68%, respectively. For lignocellulose pinewood with cobalt oxide catalyst, the syngas normalized concentrations (mole %) for H_2_, CH_4_, CO, CO_2_, and other products are 33.30, 10.76, 21.26, 33.81, and 0.85%, respectively.

The iron-based and nickel-based catalysts are used due to their cost effectiveness, which makes them an economically viable option. Additionally, iron catalysts have good thermal stability, thus enhancing their suitability for many applications. They are also capable of tolerating coking to a certain degree. During biomass gasification, iron catalysts are employed in the processes of the water–gas shift (WGS) reaction and steam reforming of hydrocarbons. These catalysts have the ability to generate a syngas that is well balanced in terms of hydrogen and carbon monoxide. We got an H_2_/CO ratio of 2.3:1. H_2_ is produced almost by doubling of CO along with CH_4_ and CO. These catalysts are well known by the name of Fischer–Tropsch catalysts. While nickel catalysts are used for their efficacy in facilitating the dry reforming of hydrocarbons and the water–gas shift (WGS) process, these catalysts have the capability to enhance the H_2_ and CO contents of the syngas and are also very helpful in significantly reducing the tar content. The utilization of nickel catalyst has been seen to result in an increase in the hydrogen and carbon monoxide concentrations within syngas, rendering it more acceptable. By utilizing Ni catalysts for the gasification process, the H_2_/CO ratio was nearly 1.1. The H_2_ produced is almost equal to CO along with CH_4_ and CO. Cobalt and molybdenum catalysts are used for their efficacy in the Fischer–Tropsch reactions. These catalysts may not directly affect the syngas composition but are vital for downstream processes like biofuel production. On the comparison of all four, we decided that iron and nickel catalysts should be selected for further and detailed study. In this study, when using both iron and nickel catalysts, we measured amounts of tar or liquid product that are too low to be detected in gas chromatography. Only traces of benzene, naphthalene, and toluene in the product stream were observed but were quantifiable using the current GC calibration, i.e., <100 ppm.

### 3.4 Biomass Catalytic Gasification in a Fixed-Bed Reactor

#### 3.4.1 Effect of Iron Catalyst

Iron-based catalysts are widely used in the synthesis of syngas from biomass gasification owing to their efficacy in facilitating the necessary chemical processes, namely, the water–gas shift (WGS) reaction and the steam reforming of hydrocarbons. While using an iron catalyst, the water–gas shift (WGS) reaction is an essential process in the creation of syngas as it facilitates the conversion of carbon monoxide (CO) and water vapor (H_2_O) into H_2_ and CO_2_. The significance of this reaction lies in its ability to augment the H_2_ content of the syngas, given that H_2_ possesses considerable value in a range of applications. The chemical reaction between CO and H_2_O can be represented as a reversible process where H_2_ and CO_2_ are produced, as shown in G8. The process of steam reforming hydrocarbons: pinewood gasification entails the presence of diverse hydrocarbons and volatile organic compounds inside the syngas. The aforementioned compounds have the capacity to undergo steam reforming processes, resulting in the generation of supplementary hydrogen.

Methane (CH_4_) reacts with water (H_2_O) in an equilibrium reaction. The chemical equation CO + 3H_2_ can be represented as C*_x_*H*_y_* + *x*H_2_O ⇌ *x*CO + (*x* + *y*/2) in a balanced form. Iron-based catalysts, converted after some time into iron oxide (Fe_3_O_4_/Fe_2_O_3_), play a crucial role in promoting these processes by offering active sites for catalytic transformations. Iron catalysts are very suitable for this purpose due to their affordability. Iron exhibits a high degree of abundance and is characterized by its comparatively low cost, rendering it a financially viable option as a catalyst when juxtaposed with noble metals such as platinum or palladium.

Iron catalysts have a notable level of catalytic activity, as they were able to effectively facilitate the water–gas shift (WGS) reaction and steam reforming under the high temperatures commonly encountered in gasification procedures. The thermal stability of iron catalysts allows them to retain their activity even under the elevated temperatures often seen during biomass gasification processes, hence potentially prolonging the operational lifespan of the catalyst. This catalyst is also suitable for this purpose due to its coking tolerance.

Iron catalysts exhibit a higher degree of carbon deposition tolerance (coking) in comparison to some other catalyst materials. The occurrence of coking in biomass gasification poses a significant issue as a result of the existence of tar and organic compounds within the feedstock. This is the reason for choosing iron due to its regeneration potential. Iron catalysts can be restored to their active state upon deactivation, a phenomenon facilitated by processes such as oxidation. This regenerative capability contributes to the elongation of their operational lifespan.^35^ Detailed information about gas, char, and tar yields from the pinewood gasification is shown in Figure S1 for high-pressure reactions and in Figure S2 for atmospheric pressure reactions.

**Figure 5:**
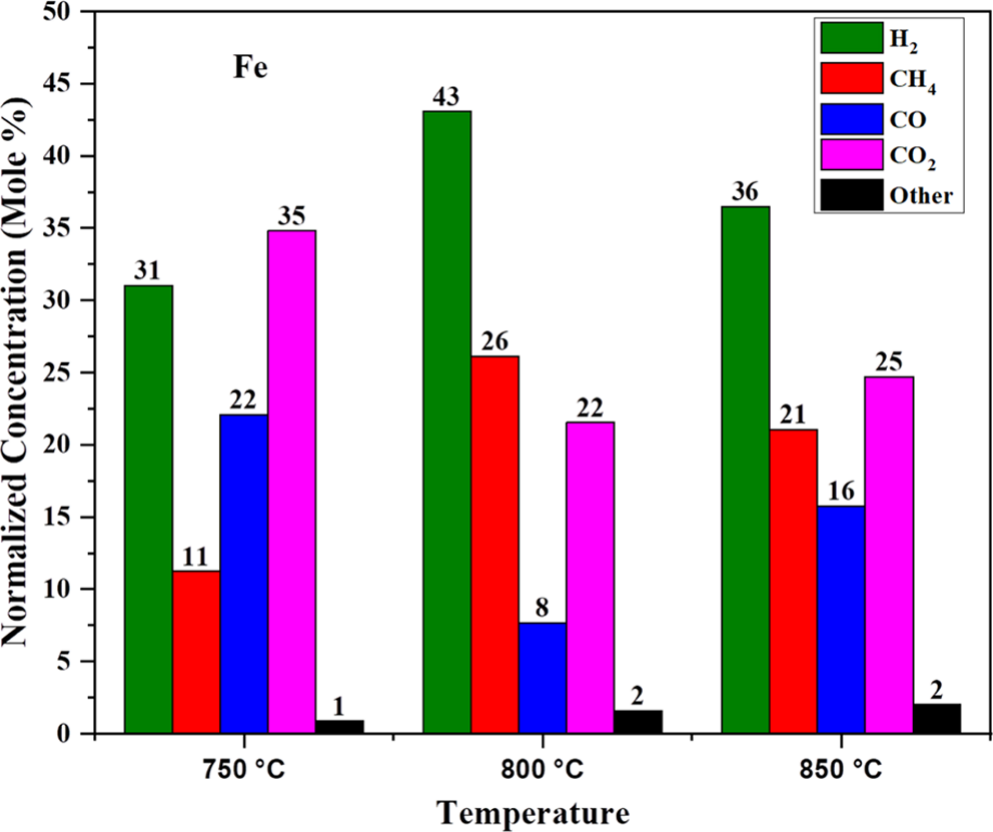
Effect of Fe on synthetic gas production with pressure.

Figure 5 shows the comparison of syngas at the different temperatures and high-pressure conditions shown in Table 2; it was observed that the concentration of H_2_ is 31 mol %, CH_4_ is 11 mol %, CO is 22 mol %, and CO_2_ is 35 mol % at 750 °C. With increase in temperature to 800 °C, the concentration of H_2_ increases in the product stream because of the heterogeneous water gas reaction G6 and also water gas shift reaction G8. Also, tar conversion occurs and is converted into gaseous form, so we conclude that CH_4_ conversion has been increased and CO conversion has been decreased due to the partial reversible water gas shift reaction G7 and we get a higher concentration of H_2_ at this temperature. On increasing the temperature to 850 °C, the H_2_ concentration decreases to 36 mol %. Due to the water gas shift reaction G8 being reversible at this temperature, tar reacts with the catalyst compounds, which get converted into oxides, increasing the concentration of CO and decreasing the concentration of H_2_.

**Figure 6:**
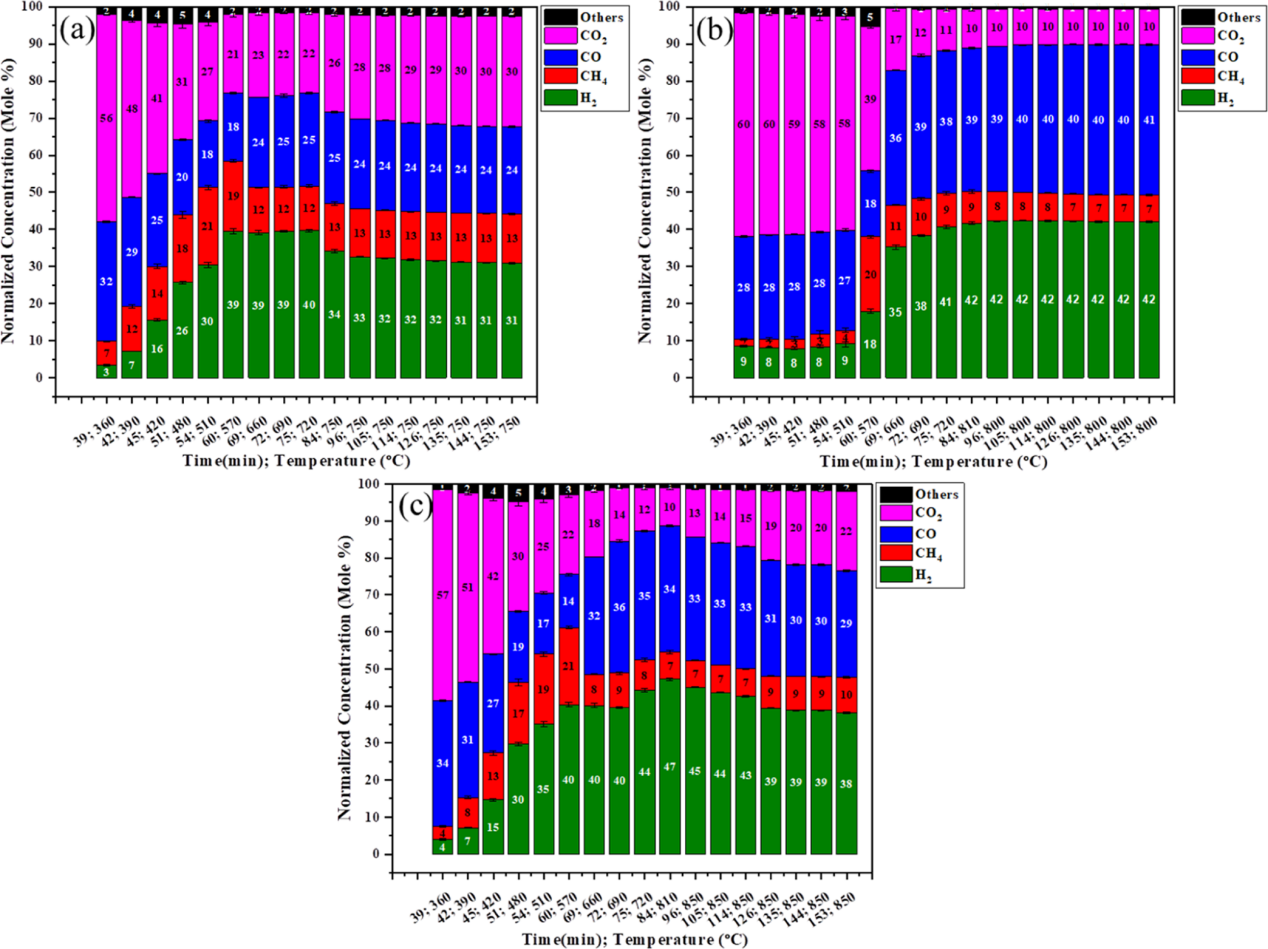
Effect of Fe with PW for synthetic gas production at low pressure and 750 °C (a), 800 °C (b), and 850 °C (c).

Figure 6a–c shows the lignocellulose biomass gasification with an iron catalyst at low pressure at different temperatures (750, 800, and 850 °C) with respect to time as shown in Table 2. When iron is used as catalyst with pinewood, it lowers the activation energy of the reaction. As shown in Figure 6a, on approaching 350 °C, the CO_2_ and CO concentrations formed are higher in comparison to using the pinewood, and also methane and hydrogen will start forming at 380 °C. After that, when the temperature increases up to 570 °C, higher H_2_ production occurs and thereafter equilibrium is achieved for the WGS reaction; following this, the tar reaction takes place and more CO_2_ is formed; this reaction is reversible, so H_2_ is converted to CO again. Thus, the H_2_ concentration again decreases to a lower content after 720 °C. As shown in Figure 6b, CO_2_ and CO concentrations are higher at 350 °C and CH_4_ and H_2_ concentrations are lower; after that, the water gas shift reaction G8 happens, which increases the main rate of the reaction and also tar conversion occurs into a gaseous product. Equilibrium is achieved and we observe the maximum concentration of syngas at this temperature after 105 min. The syngas concentration comprises 42.324 mol % H_2_, 7.74 mol % CH_4_, 39.66 mol % CO, 9.75 mol % CO_2_, and 0.55 mol % others. At 850 °C, the H_2_ concentration is increased due to some tar conversion reactions taking place at high temperature using the Fe catalyst. Meanwhile, some char reacts with H_2_ and forms CH_4_ after 800 °C in G4. Here, the dry reforming reaction G14 is partially reversible, which decreases the concentration of CO and increases the concentration of CO_2_. The syngas concentration comprises 47.18 mol % H_2_, 7.3 mol % CH_4_, 34.2 mol % CO, 10.274 mol % CO_2_, and 0.99 mol % others after 84 min at 810 °C.

#### 3.4.2 Effect of Nickel Catalyst

Nickel catalysts are widely utilized in the synthesis of syngas from biomass gasification due to their effectiveness in stimulating critical processes such as hydrocarbon steam reforming and the WGS reaction. These catalysts exhibit favorable compatibility with specific gasification conditions and possess numerous merits that make them a suitable option for the generation of syngas from biomass. The process of steam reforming hydrocarbons involves the production of a gas mixture by biomass gasification, which might potentially contain hydrocarbons such as CH_4_ and volatile organic compounds (VOCs). Nickel catalysts exhibit high efficacy in facilitating steam reforming processes, which facilitate the conversion of hydrocarbons into syngas constituents, namely, CO and H_2_. Enhancing the hydrogen concentration of syngas holds significant importance.^36,37^ Methane (CH_4_) reacts with water (H_2_O) in an equilibrium reaction. The chemical equation CO + 3H_2_ can be represented as C*_x_*H*_y_* + *x*H_2_O ⇌ *x*CO + (*x* + *y*/2)H_2_. The WGS reaction is known to play a crucial role in promoting this reaction, which is a chemical process that transforms CO and water vapor (H_2_O) into H_2_ and CO_2_. The reaction represented by CO + H_2_O ⇌ H_2_ + CO_2_ is of interest in the context of biomass gasification similar to the Fe catalyst, which is discussed above. One typical obstacle in this process is the deposition of carbon, often known as coking. However, nickel catalysts have been found to have a high tolerance to carbon deposition. This is particularly advantageous given the presence of tar and organic compounds in the biomass feedstock. Nickel exhibits notable catalytic activity when subjected to the higher temperatures commonly encountered in gasification processes.^36^ The detailed information on gas, char, and tar yield from the pinewood gasification is shown in Figure S1 for high-pressure reactions and in Figure S2 for atmospheric pressure reactions.

**Figure 7:**
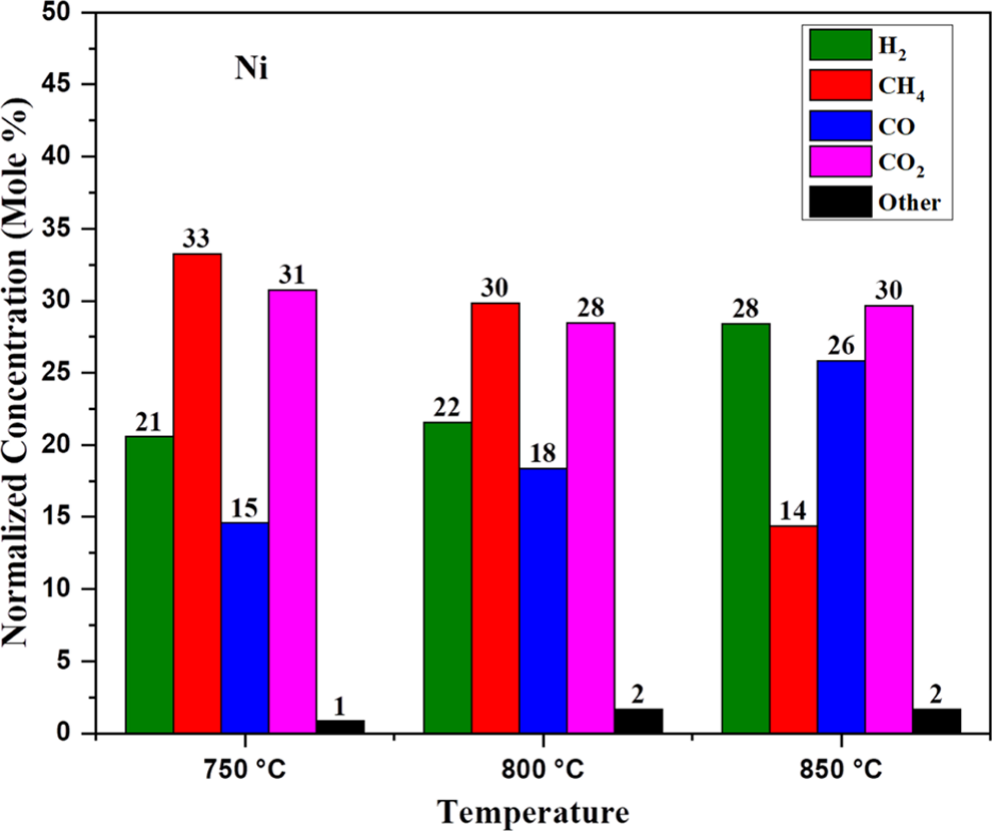
Effect on Ni for synthetic gas production with pressure.

Figure 7 shows that nickel catalyst added to the pinewood lowers the activation energy of the system during gasification reaction and nickel catalyst also helps in tar conversion of the products into gaseous form like in CO, H_2_, CO_2_, and CH_4_. At 750 °C, we observed 21 mol % H_2_, 33 mol % CH_4_, 15 mol % CO, 31 mol % CO_2_, and 1 mol % others, and after increasing the temperature to 800 °C, we observed that some cracking reaction occurs because the CH_4_ concentration has been decreased and also CO concentration has been decreased. At 850 °C, the CH_4_ concentration decreases as the CO concentration increases due to steam reforming of methane reaction, which is G7 and reversible to some extent. The detailed syngas composition is shown in Table 2.

**Figure 8:**
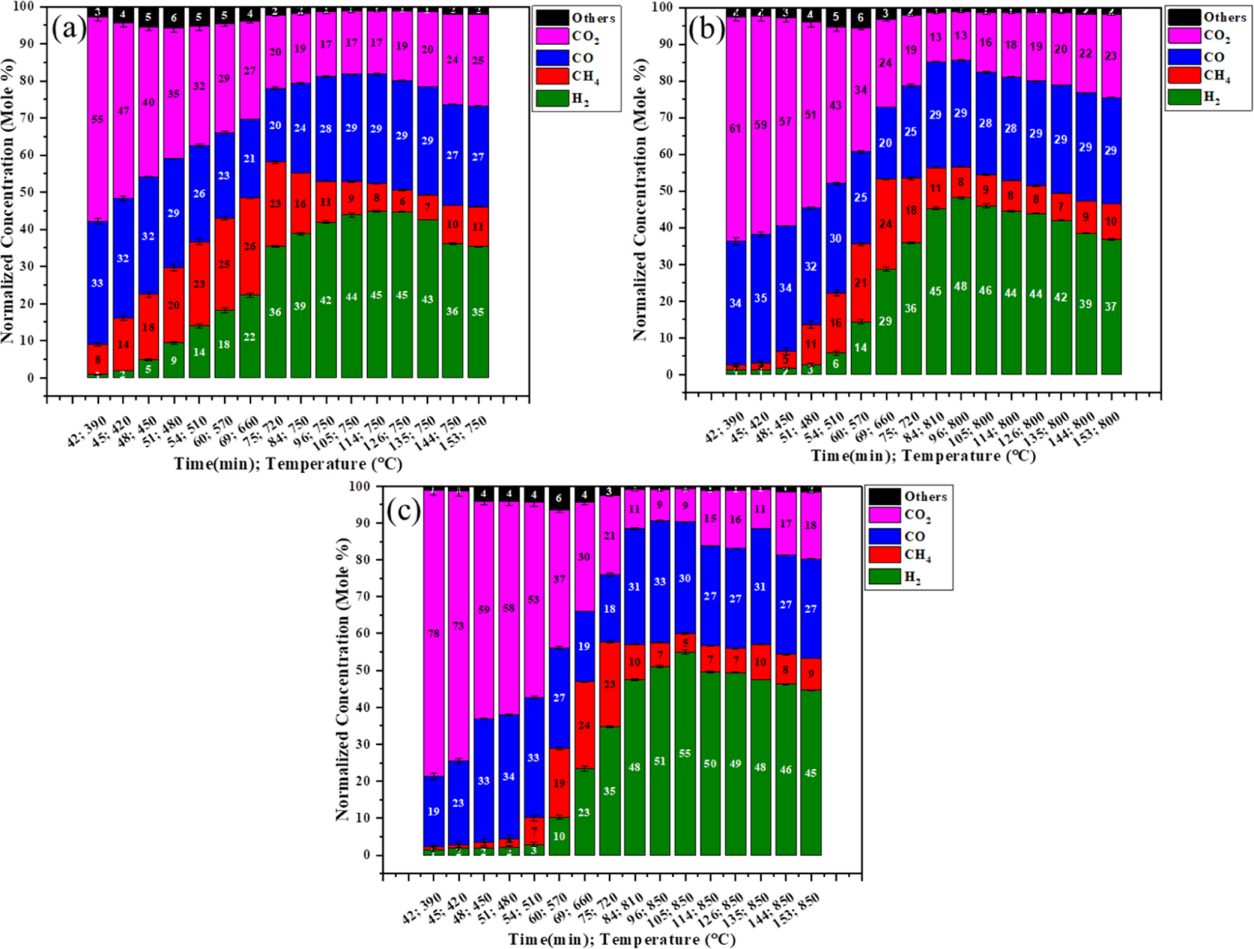
Effect of Ni with PW for synthetic gas production at low pressure and 750 °C (a), 800 °C (b), and 850 °C (c).

Figure 8a–c shows the lignocellulose biomass gasification with nickel catalyst at low pressure at different temperatures (750, 800, and 850 °C) with respect to time as shown in Table 2. The concentration profiles with time on stream data using the nickel catalyst with pinewood at low temperatures are shown. Initially, we observe in Figure 8a that CO_2_ and CO are formed at a maximum of around 330 °C and on increasing the temperature, the concentrations of CO_2_ and CO decrease due to the water gas shift section reaction G8 and char gasification reactions G4–G6, which increase the concentrations of H_2_ and CO again. Thereafter, at 850 °C, we observe that initially more combustion reaction happens than the previous temperatures (750 and 800 °C) due to the catalyst lowering the activation energy, CO_2_ and CO are converted into hydrogen, and the concentration increases significantly and thereafter decreases to some extent due to the water gas shift reaction G8. In parallel, the CO concentration is decreased and then again increases and CH_4_ concentration is decreased; due to steam methane reforming, it converts into CO and H_2_ and here we observe the highest concentration of H_2_ using nickel catalyst at 850 °C. The syngas concentration comprises 44.8 mol % H_2_, 7.5 mol % CH_4_, 29.4 mol % CO, 17.06 mol % CO_2_, and 1.12 mol % others at 750 °C after 114 min and the syngas concentration at 800 °C is 48.09 mol % H_2_, 8.48 mol % CH_4_, 29.1 mol % CO, 13.28 mol % CO_2_, and 1.03 mol % others at 800 °C after 96 min. The syngas concentration is 54.98 mol % H_2_, 4.9 mol % CH_4_, 30.3 mol % CO, 9.1 mol % CO_2_ and 0.65 mol % others at 850 °C after 105 min.

### 3.5 Pressure Effects Comparison on Gasification in a Fixed-Bed Reactor

**Figure 9:**
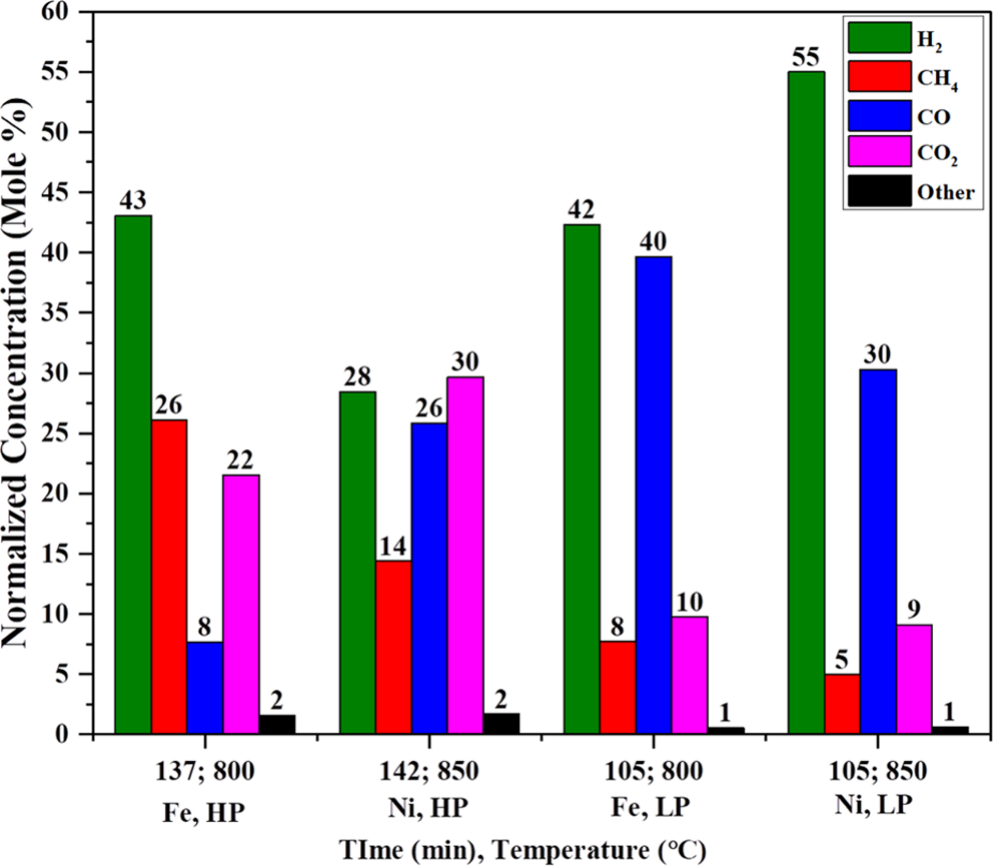
Effect of pressure on Fe and Ni catalysts.

There are only a few studies reported for low pressures either with a low gasifying agent (0–5%) or without the gasifying agent as shown in Table 3. Table 3 shows the comparison of previous studies with this study. Figure 9 shows the highest conversion of syngas during catalytic gasification of iron and nickel catalysts at high pressure and low pressure. We can conclude that when the reaction happens between 28 and 38 bar, the iron catalyst gives the highest concentration of syngas along with a large amount of methane, containing 43 mol % H_2_, 22 mol % CO_2_, 26 mol % CH_4_, 8 mol % CO, and 2 mol % others at 800 °C and 137 min in comparison to syngas concentration using nickel catalyst, which gives 28 mol % H_2_, 26 mol % CO_2_, 14 mol %, 26 mol % CO, and 2 mol % others at 850 °C and 142 min. At high pressure, we get a pretty good amount of methane in syngas due to hydrogenation reaction G4 in the case of using nickel catalyst. Hydrogenation reaction G4 increases the rate of reaction using an iron catalyst in comparison to a nickel catalyst in high-pressure reaction. On gasification using Ni catalyst, steam reforming of methane G7 and oxidation of methane reaction G10 take place for increasing the rate of reaction with water gas shift reaction G8, which act as partially reversible too for a higher amount of production of syngas. This reaction happens to only such extent due to water vapors and oxygen acting as limiting reactants.

**Table 3:** Comparison of Syngas Production from Lignocellulose Pinewood

			gas composition					
catalyst	temperature (°C)	syngas yield^a^	CO_2_	CO	H_2_	CH_4_	others	refs
ilemenite, FeTiO_3_	820	0.34 (Nm^3^/kg biomass)	41.8 (vol %)	22.7 (vol %)	18.4 (vol %)	13 (vol %)	4.1 (vol %)	(28)
ilemenite, FeTiO_3_	880	0.41 (Nm^3^/kg biomass)	44.9 (vol %)	21.7 (vol %)	18.9 (vol %)	12.0 (vol %)	2.5 (vol %)	
ilemenite, FeTiO_3_	940	0.55 (Nm^3^/kg biomass)	33.4 (vol %)	29.2 (vol %)	25 (vol %)	10.8 (vol %)	1.6 (vol %)	
20 wt % Fe_2_O_3_/Al_2_O_3_	940	0.74 (Nm^3^/kg biomass)	25 (vol %)	37 (vol %)	32 (vol %)	9.5 (vol %)	1 (vol %)	(29)
CaO + sand	780		30.2 (vol %)	34.9 (vol %)	21.1 (vol %)	9.7 (vol %)	4.1 (vol %)	(30)
Al_2_O_3_	780		28.7 (vol %)	38.2 (vol %)	14.7 (vol %)	11.8 (vol %)	6.5 (vol %)	
CaO + Al_2_O_3_	780		29.7 (vol %)	36.1 (vol %)	21.3 (vol %)	9.3 (vol %)	3.5 (vol %)	
Fe	800 (HP)	0.88 (g mol/g biomass)	7.67 (mol %)	21.54 (mol %)	43.07 (mol %)	26.13 (mol %)	1.59 (mol %)	this study
Ni	850 (LP)	0.89 (g mol/g biomass)	30.30 (mol %)	9.10 (mol %)	54.99 (mol %)	4.95 (mol %)	0.65 (mol %)	

a Yield is the ratio of moles of the desired product (syngas) to the initial moles of the reactant (biomass).

Actually the highest syngas production, we were observed that low-pressure reaction at 1.01 bar, nickel catalyst gives the highest concentration of syngas along with low amount of CH_4_ and CO_2_ contains 55 mol % H_2_, 9 mol % CO_2_, 30 mol % CO, 5 mol % CO_2_ and 1 mol % others at 850 °C and after 105 min in comparison to syngas concentration using iron catalyst gives 42 mol % H_2_, 10 mol % CO_2_, 40 mol %, 8 mol % CO and 1 mol % others at 800 °C after 105 min.

At low pressure, we observed low amounts of CH_4_ and CO_2_ with high amounts of syngas due to hydrogenation reaction G4. Hydrogenation reaction G4, water gas shift reaction G8 and steam reforming of methane G7 increases the rate of reaction for both catalysts. When gasification using Ni catalyst, we are getting 23 mol % more H_2_ due to heterogeneous water gas reaction G6, water gas shift reaction G8 and reverse Boudouard reaction G5 increases the rate of reaction. One more interesting thing we observed that H_2_/CO ration is half during high pressure and H_2_/CO ratio close to 1 at low pressure using iron catalyst and fully reverse in case of using nickel catalyst.

### 3.6 Char Analysis

The characterization of char in pinewood gasification is contingent upon the particular gasification parameters, which encompass factors, such as pressure. The process of gasification under high pressure has been seen to produce chars that exhibit distinct properties in comparison to those generated during gasification under atmospheric pressure. Gasification under high pressure, such as supercritical water gasification, has a notable impact on the production and characteristics of char. Under increased pressures, the reaction kinetics and equilibrium conditions undergo changes, potentially impacting the composition of the char. Under low pressure, the gasification conditions are comparatively less severe, resulting in potential variations in the properties of char compared to gasification under high pressure.

The results obtained from C, H, N, and S analysis also indicate 60–70% carbon present in char in high pressurized reactions and 30–40% carbon in char content at low pressures. It was observed that the use of a high pressure during gasification procedures significantly affects the production of char. High-pressure circumstances, in particular, were observed to facilitate the generation of char with a higher graphitic concentration and lower reactivity. The reason for this impact is that high-pressure levels cause secondary reactions to occur more quickly. This phenomenon has the potential to result in a type of char that possesses an elevated carbon content. At atmospheric pressure, the composition of char may exhibit an increased proportion of volatile materials, resulting in heightened reactivity and possible suitability for various uses such as the manufacture of activated carbon.

## 4 Conclusions

Gasification of pinewood biomass was conducted with and without a catalyst. The results show that with incorporation of metals (Fe, Mo, CoO, Ni) an increase in H_2_ production with 36, 34, 33, and 28 mol % was observed, respectively, at a high pressure and 850 °C. The efficiency of syngas production utilizing iron and nickel catalysts was investigated over several different conditions. At higher pressures (800 °C), the iron catalyst performed better, producing syngas with 43 mol % H_2_, 22 mol % CO_2_, 26 mol % CH_4_, and 8 mol % CO. Notably, the high-pressure condition enabled considerable methane synthesis along with syngas with a nickel catalyst. At lower pressure and 850 °C, however, the nickel catalyst produced more syngas, including 55 mol % H_2_, 9 mol % CO_2_, 5 mol % CH_4_, and 30 mol % CO. The highest H_2_:CO ratio of 5.4% (based on mol %) was observed for the Fe-based catalyst at 800 °C reaction temperature and 35 bar pressure. The high yield of H_2_ was observed due to the oxophilic nature of Fe, which converts the oxygen content in lignin into steam, which at high temperature reacts with the produced methane to form hydrogen. An intriguing finding was observed throughout the syngas production process about the influence of reaction pressure and catalyst type. A nickel catalyst is the most effective at low pressure (around 1.01 bar), providing the maximum concentration of syngas. At 850 °C, this nickel catalyst-produced syngas had minimal methane and carbon monoxide content, as well as an excellent hydrogen content of 55 mol %.

## Supporting Information Available

Description of graphical abstract; detailed information on gas; char and tar yield of lignocellulose pinewood of noncatalytic and catalytic gasification reactions at high and low pressures; characterization discussion (XRD, SEM) of pinewood and catalyst (PDF)
