# Supplementary material for: Unlocking Syngas Synthesis from the Catalytic Gasification of Lignocellulose Pinewood: Catalytic and Pressure Insights
Source: ACS Sustain Chem Eng. 2024 Mar 7;12(11):4718–30. doi: 10.1021/acssuschemeng.4c00320 (PMC10952009; doi:10.1021/acssuschemeng.4c00320)
Supplement: Supplementary file 1 — sc4c00320_si_001.pdf [file sc4c00320_si_001.pdf]

Unlocking Syngas Synthesis from the Catalytic Gasification of Lignocellulose Pinewood: Catalytic and Pressure Insights

Kshitij Tewari, Sonit Balyan, Changle Jiang, Brandon Robinson, Debangsu Bhattacharyya, Jianli Hu*

Department of Chemical and Biomedical Engineering, West Virginia University, Morgantown, West Virginia 26506, United States

*Corresponding Author: john.hu@mail.wvu.edu

This supporting information contains:

- No of Pages: 5 pages (S1-S7),
- One Supporting Table (S1)
- Four Supporting Figures (Figures S1-S4)

Summary

S. No.	Description	Page No.
1	Graphical abstract description	S2
2	Figure S1: Gas, Char, and Tar Yield of pinewood gasification at high pressures (High pressures)	S3
3	Figure S2: Gas, Char, and Tar Yield of pinewood gasification at high pressures (Atmospheric pressures)	S4
4	Figure S3: SEM (a, b) Pinewood Char (c) Iron catalyst (d) Iron Char (e) Nickel catalyst (f) Nickel Char (Temperature: 850 °C)	S5
5	Figure S4: XRD of lignocellulose pinewood (PW), catalyst (Fe, Ni , Mo, CoO) and Fe-HP, Ni-LP gasification (Temperature: 850 °C)	S6
6	Table S1: Various catalyst characteristics	S7

Graphical abstract illustrates the C−O bond types in lignin depicted by the symbol R, representing an aromatic structure akin to the rest of the proposed description of the lignin. It depicts the structure of aromatic and furfural chains in lignin, including −OH functional groups. The surface's weakly linked oxygen and hydroxyl groups have the ability to react with hydrogen produced by biomass devolatilization gases. This reaction causes the formation of H_2O adsorbates on the active sites of Ni and Fe. This structural understanding implies a hydrogen generating pathway involving interactions between lignin components and metal catalysts during biomass processing.

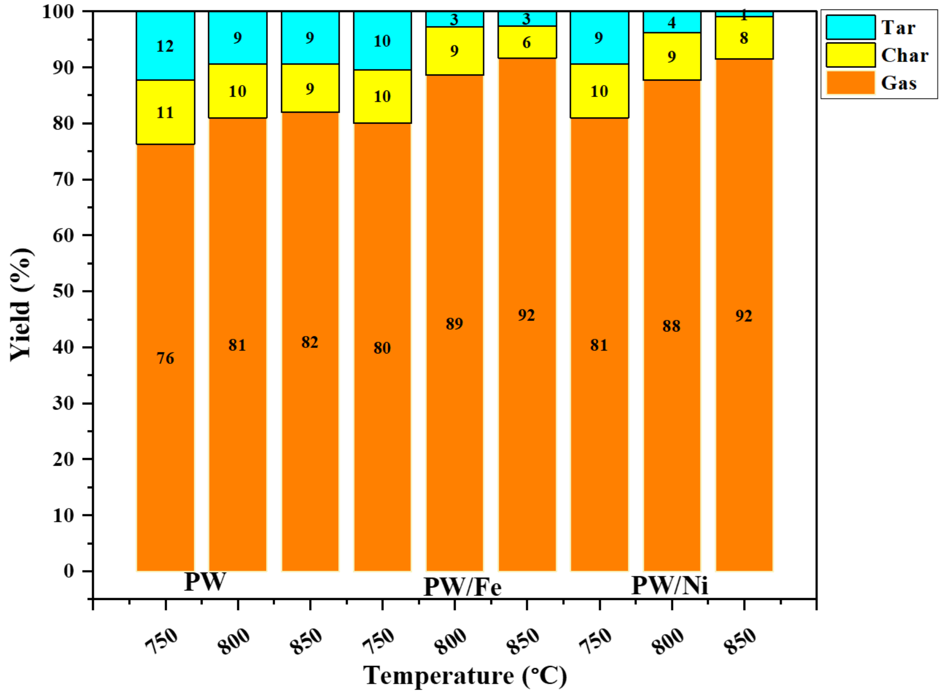

Figure S1: Gas, Char, and Tar Yield of pinewood gasification at high pressures (High pressures)

Figure S1 represents the detailed information of gas, char, and tar yield at various temperatures 750, 800, and 850 °C with non-catalytic gasification has pinewood only, and the other two are catalytic gasification with iron and nickel catalysts at high pressures.

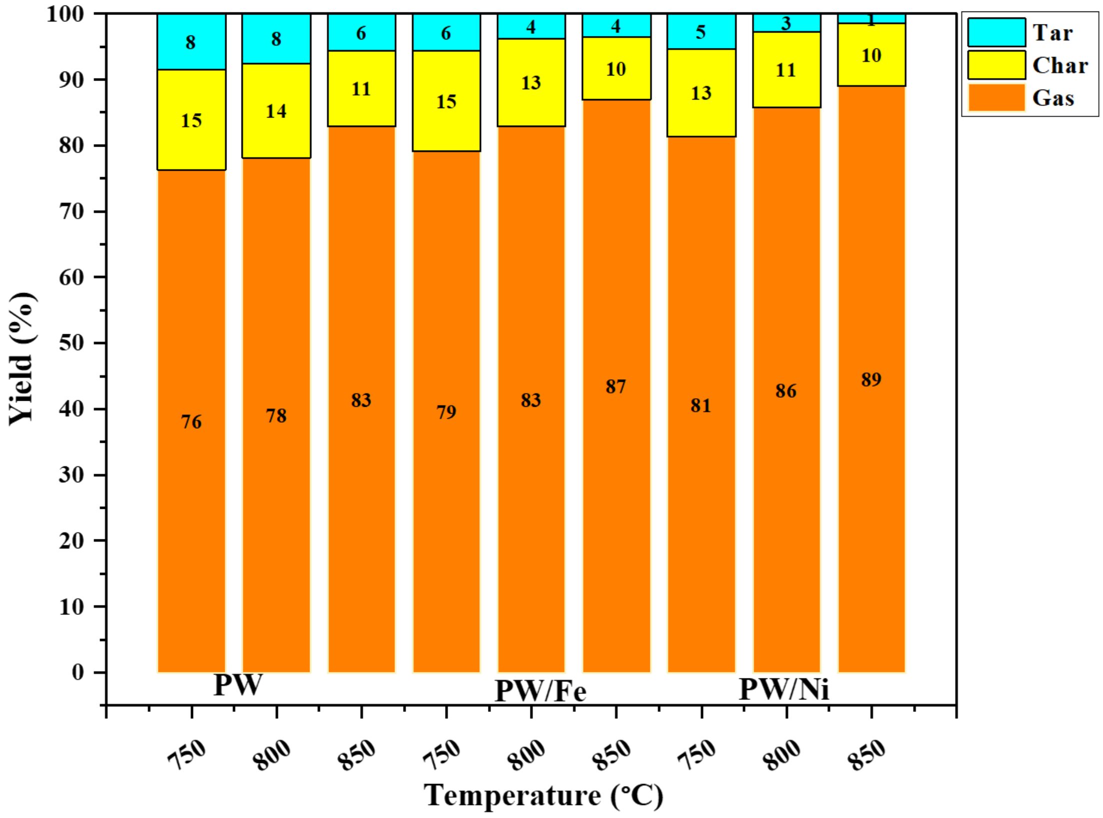

Figure S2: Gas, Char, and Tar Yield of pinewood gasification at high pressures (Atmospheric pressures)

Figure S2 represents the detailed information of gas, char, and tar yield at various temperatures 750, 800, and 850 °C with non-catalytic gasification has pinewood only, and the other two are catalytic gasification with iron and nickel catalysts at atmospheric pressures.

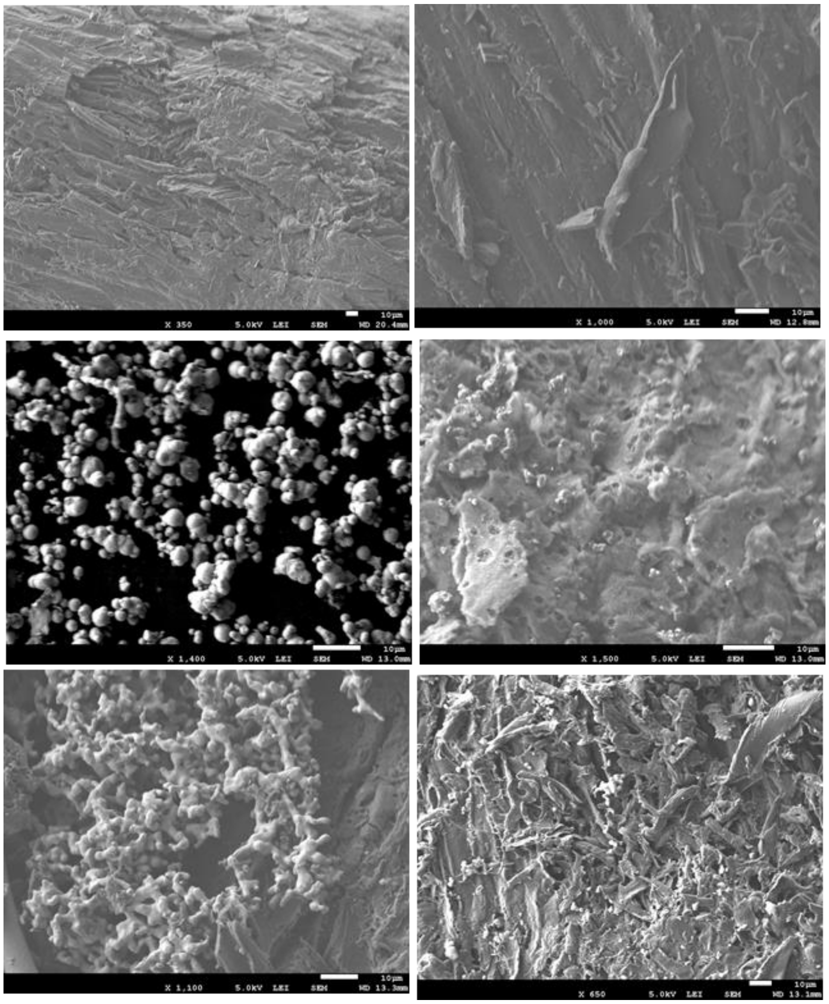

Figure S3: SEM (a, b) Pinewood Char (c) Iron catalyst (d) Iron Char (e) Nickel catalyst (f) Nickel Char (Temperature: 850 °C)

Figures S3 a, c, and e show the lignocellulose pinewood, iron, and nickel catalyst morphology, respectively. In c and e, we can see the shiny part, which is the iron and nickel catalyst, respectively, in SEM images. Fig b, d and e are images after gasification. Fig a is lignocellulose pinewood only, which shows the char morphology at 850 °C. The other two, Fig. d and f, show the morphology of pinewood with iron and nickel catalyst after gasification at 850 °C in which metal is clearly visible with char.

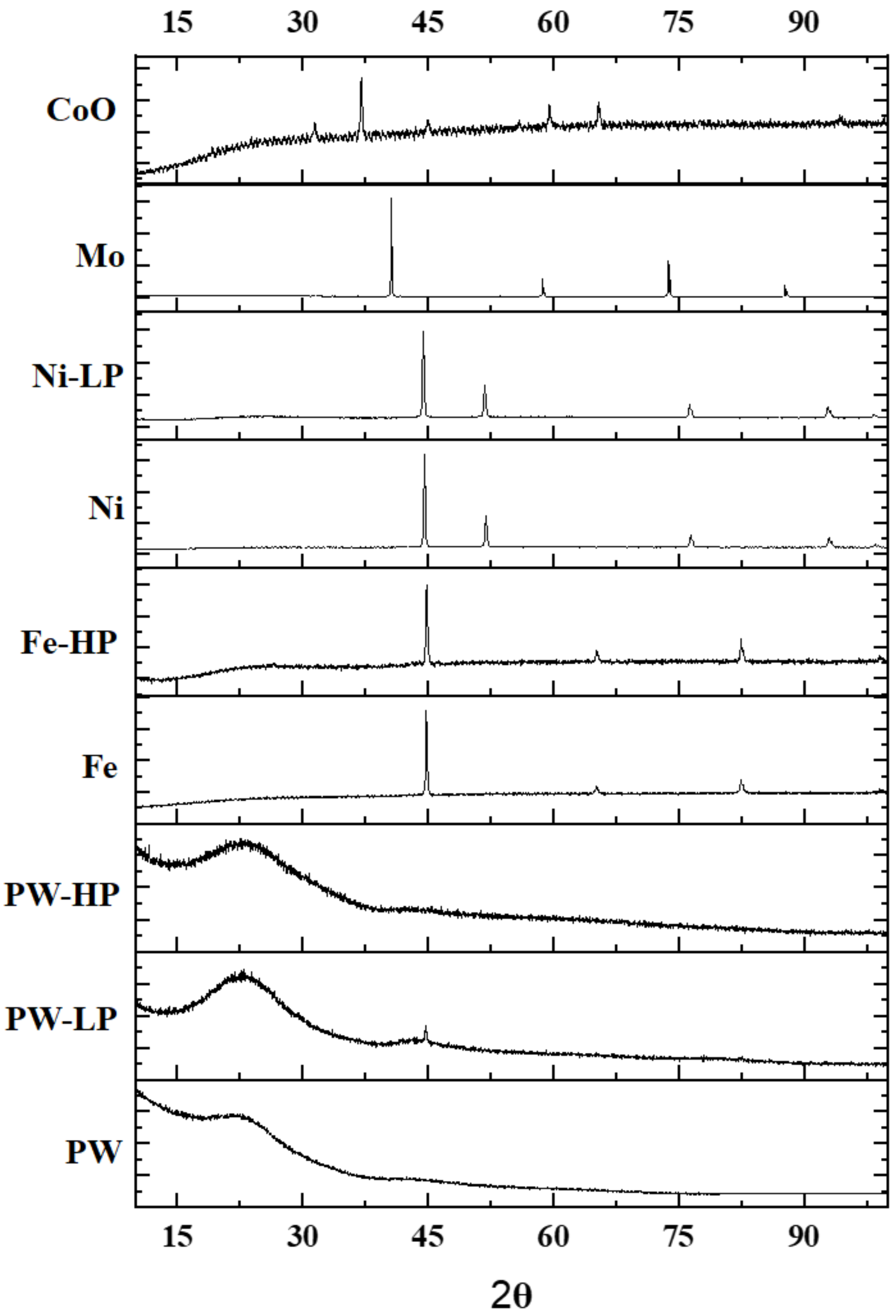

Figure S4: XRD of lignocellulose pinewood (PW), catalyst (Fe, Ni , Mo, CoO) and Fe-HP, Ni-LP gasification (Temperature: 850 °C)

Figure S4 shows the XRD analysis of raw material lignocellulose pinewood and various catalysts used in the study, such as Fe, Ni, Mo, and CoO, respectively. These four-catalysts prepared well confirmed by JCPDS. All the peaks' data of each catalyst match. Iron catalyst peaks confirmed by JCPDS#00-006-0696. Nickel catalyst peaks confirmed by JCPDS# 03-065-

2865. Molybdenum catalyst peaks confirmed by JCPDS# 00-042-1120. Cobalt oxide catalyst peaks confirmed by JCPDS# 00-025-0270. Also, we reported XRD of the iron catalytic gasification with PW at high pressure and nickel catalytic gasification with PW at low pressure. These two reactions produce higher syngas production at high pressure and low pressures. Also, we observed that in these two XRDs, we found peaks of a mixture of metal and metal oxide.

Table S1: Various catalyst characteristics

Catalyst	BET surface area	Avg Particle Size
Fe	0.66 m^2/g	9,098.39 nm
Ni	0.75 m^2/g	7,975.72 nm
Mo	0.84 m^2/g	7,144.93 nm
CoO	2.22 m^2/g	2,707.80 nm
